# 4-Ethyl-1-(4-methyl­benzyl­idene)thio­semicarbazide

**DOI:** 10.1107/S1600536810017988

**Published:** 2010-05-22

**Authors:** Yu-Feng Li, Fang-Fang Jian

**Affiliations:** a Microscale Science Institute, Department of Chemistry and Chemical Engineering, Weifang University, Weifang 261061, People’s Republic of China; b Microscale Science Institute, Weifang University, Weifang 261061, People’s Republic of China

## Abstract

In the title compound, C_11_H_15_N_3_S, an intra­molecular N—H⋯N hydrogen bond generates an *S*(5) ring. In the crystal, inversion dimers linked by pairs of N—H⋯S bonds occur, generating an *R*_2_^2^(8) loop.

## Related literature

For a related structure, see: Li & Jian (2010 ▶).
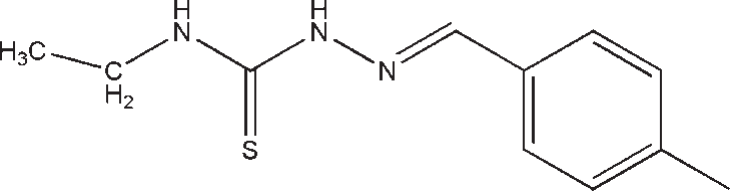


## Experimental

### 

#### Crystal data

C_11_H_15_N_3_S
*M* *_r_* = 221.32
Monoclinic, 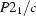

*a* = 8.5777 (17) Å
*b* = 13.620 (3) Å
*c* = 10.364 (2) Å
β = 90.00 (3)°
*V* = 1210.8 (4) Å^3^
*Z* = 4
Mo *K*α radiation
μ = 0.24 mm^−1^
*T* = 293 K
0.23 × 0.20 × 0.18 mm

#### Data collection

Bruker SMART CCD diffractometer
11225 measured reflections
2724 independent reflections
1811 reflections with *I* > 2σ(*I*)
*R*_int_ = 0.035

#### Refinement

*R*[*F* ^2^ > 2σ(*F* ^2^)] = 0.045
*wR*(*F* ^2^) = 0.144
*S* = 1.10
2724 reflections
136 parameters
H-atom parameters constrained
Δρ_max_ = 0.22 e Å^−3^
Δρ_min_ = −0.21 e Å^−3^

### 

Data collection: *SMART* (Bruker, 1997 ▶); cell refinement: *SAINT* (Bruker, 1997 ▶); data reduction: *SAINT*; program(s) used to solve structure: *SHELXS97* (Sheldrick, 2008 ▶); program(s) used to refine structure: *SHELXL97* (Sheldrick, 2008 ▶); molecular graphics: *SHELXTL* (Sheldrick, 2008 ▶); software used to prepare material for publication: *SHELXTL*.

## Supplementary Material

Crystal structure: contains datablocks global, I. DOI: 10.1107/S1600536810017988/hb5448sup1.cif

Structure factors: contains datablocks I. DOI: 10.1107/S1600536810017988/hb5448Isup2.hkl

Additional supplementary materials:  crystallographic information; 3D view; checkCIF report

## Figures and Tables

**Table 1:** Hydrogen-bond geometry (Å, °)

*D*—H⋯*A*	*D*—H	H⋯*A*	*D*⋯*A*	*D*—H⋯*A*
N2—H2*A*⋯S1^i^	0.86	2.69	3.505 (2)	158
N3—H3*A*⋯N1	0.86	2.21	2.605 (2)	108

Symmetry code: (i) 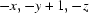.

**Fig. 1.**
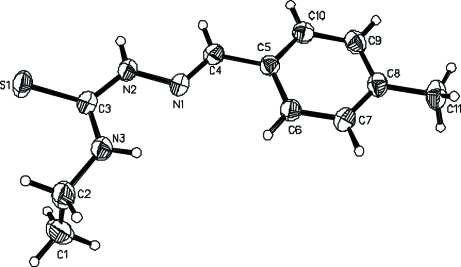
The structure of (I) showing 30% probability displacement ellipsoids.

## supplementary crystallographic information

### Experimental

A mixture of 4-methylbenzaldehyde (0.10 mol), and 4-ethylthiosemicarbazide (0.10 mol) was stirred in refluxing ethanol (10 ml) for 4 h to afford the title compound (0.078 mol, yield 78%).  Colourless blocks of (I) were obtained by recrystallization from ethanol at room temperature.

### Refinement

H atoms were fixed geometrically and allowed to ride on their attached atoms, with C—H distances = 0.93-0.97 Å; N—H = 0.86Å and with *U*_iso_(H) = 1.2*U*_eq_(C,*N*) or 1.5*U*_eq_(C_methyl_).

### Crystal data

**Table:**

C_11_H_15_N_3_S	*F*(000) = 472
*M**_r_* = 221.32	*D*_x_ = 1.214 Mg m^−^^3^
Monoclinic, *P*2_1_/*c*	Mo *K*α radiation, λ = 0.71073 Å
Hall symbol: -P 2ybc	Cell parameters from 1811 reflections
*a* = 8.5777 (17) Å	θ = 3.7–25.4°
*b* = 13.620 (3) Å	µ = 0.24 mm^−^^1^
*c* = 10.364 (2) Å	*T* = 293 K
β = 90.00 (3)°	Block, colorless
*V* = 1210.8 (4) Å^3^	0.23 × 0.20 × 0.18 mm
*Z* = 4	

### Data collection

**Table:**

Bruker SMART CCD diffractometer	1811 reflections with *I* > 2σ(*I*)
Radiation source: fine-focus sealed tube	*R*_int_ = 0.035
graphite	θ_max_ = 27.5°, θ_min_ = 3.4°
phi and ω scans	*h* = −10→11
11225 measured reflections	*k* = −17→17
2724 independent reflections	*l* = −13→12

### Refinement

**Table:**

Refinement on *F*^2^	Primary atom site location: structure-invariant direct methods
Least-squares matrix: full	Secondary atom site location: difference Fourier map
*R*[*F*^2^ > 2σ(*F*^2^)] = 0.045	Hydrogen site location: inferred from neighbouring sites
*wR*(*F*^2^) = 0.144	H-atom parameters constrained
*S* = 1.10	*w* = 1/[σ^2^(*F*_o_^2^) + (0.0722*P*)^2^ + 0.0957*P*] where *P* = (*F*_o_^2^ + 2*F*_c_^2^)/3
2724 reflections	(Δ/σ)_max_ = 0.001
136 parameters	Δρ_max_ = 0.22 e Å^−^^3^
0 restraints	Δρ_min_ = −0.21 e Å^−^^3^

### Special details

**Table:**

Geometry. All esds (except the esd in the dihedral angle between two l.s. planes) are estimated using the full covariance matrix. The cell esds are taken into account individually in the estimation of esds in distances, angles and torsion angles; correlations between esds in cell parameters are only used when they are defined by crystal symmetry. An approximate (isotropic) treatment of cell esds is used for estimating esds involving l.s. planes.
Refinement. Refinement of *F*^2^ against ALL reflections. The weighted *R*-factor wR and goodness of fit *S* are based on *F*^2^, conventional *R*-factors *R* are based on *F*, with *F* set to zero for negative *F*^2^. The threshold expression of *F*^2^ > σ(*F*^2^) is used only for calculating *R*-factors(gt) etc. and is not relevant to the choice of reflections for refinement. *R*-factors based on *F*^2^ are statistically about twice as large as those based on *F*, and *R*- factors based on ALL data will be even larger.

### Fractional atomic coordinates and isotropic or equivalent isotropic displacement parameters (Å^2^)

**Table:**

	*x*	*y*	*z*	*U*_iso_*/*U*_eq_	
S1	0.18659 (7)	0.61350 (4)	0.05705 (5)	0.0646 (2)	
N2	0.02730 (19)	0.59199 (13)	−0.15745 (14)	0.0517 (4)	
H2A	−0.0314	0.5527	−0.1144	0.062*	
N1	−0.00273 (18)	0.61045 (12)	−0.28521 (14)	0.0471 (4)	
C5	−0.1597 (2)	0.57835 (14)	−0.47004 (17)	0.0454 (4)	
C6	−0.0800 (2)	0.64307 (15)	−0.55008 (18)	0.0514 (5)	
H6A	0.0041	0.6785	−0.5179	0.062*	
C8	−0.2494 (2)	0.60447 (16)	−0.72820 (18)	0.0535 (5)	
C4	−0.1162 (2)	0.56386 (15)	−0.33565 (18)	0.0494 (5)	
H4A	−0.1724	0.5196	−0.2855	0.059*	
C3	0.1500 (2)	0.63616 (15)	−0.09996 (17)	0.0486 (5)	
N3	0.2320 (2)	0.69703 (14)	−0.17353 (16)	0.0586 (5)	
H3A	0.1966	0.7095	−0.2494	0.070*	
C7	−0.1245 (2)	0.65502 (17)	−0.67644 (18)	0.0556 (5)	
H7A	−0.0691	0.6983	−0.7285	0.067*	
C10	−0.2838 (2)	0.52628 (17)	−0.52182 (19)	0.0582 (5)	
H10A	−0.3384	0.4819	−0.4706	0.070*	
C9	−0.3270 (2)	0.53994 (18)	−0.6490 (2)	0.0618 (6)	
H9A	−0.4107	0.5045	−0.6819	0.074*	
C11	−0.2989 (3)	0.6213 (2)	−0.8660 (2)	0.0747 (7)	
H11A	−0.3868	0.5803	−0.8857	0.112*	
H11B	−0.2142	0.6054	−0.9228	0.112*	
H11C	−0.3272	0.6890	−0.8774	0.112*	
C2	0.3779 (3)	0.7445 (2)	−0.1356 (2)	0.0759 (7)	
H2B	0.3760	0.7574	−0.0436	0.091*	
H2C	0.3869	0.8071	−0.1798	0.091*	
C1	0.5171 (3)	0.6822 (2)	−0.1673 (3)	0.0875 (9)	
H1B	0.6104	0.7152	−0.1403	0.131*	
H1C	0.5210	0.6710	−0.2587	0.131*	
H1D	0.5089	0.6204	−0.1232	0.131*	

### Atomic displacement parameters (Å^2^)

**Table:**

	*U*^11^	*U*^22^	*U*^33^	*U*^12^	*U*^13^	*U*^23^
S1	0.0854 (4)	0.0693 (4)	0.0392 (3)	−0.0124 (3)	−0.0082 (2)	0.0033 (2)
N2	0.0574 (10)	0.0600 (11)	0.0377 (8)	−0.0058 (8)	−0.0014 (7)	0.0049 (7)
N1	0.0559 (9)	0.0484 (9)	0.0372 (8)	0.0019 (7)	−0.0015 (7)	0.0003 (6)
C5	0.0449 (10)	0.0467 (11)	0.0445 (9)	0.0024 (8)	−0.0011 (7)	−0.0003 (7)
C6	0.0552 (11)	0.0497 (11)	0.0493 (10)	−0.0063 (9)	−0.0042 (8)	0.0026 (8)
C8	0.0596 (12)	0.0557 (12)	0.0451 (10)	0.0138 (10)	−0.0061 (9)	−0.0064 (8)
C4	0.0509 (11)	0.0510 (12)	0.0463 (10)	−0.0008 (9)	0.0016 (8)	0.0047 (8)
C3	0.0561 (11)	0.0490 (11)	0.0406 (9)	0.0019 (9)	−0.0004 (8)	−0.0009 (8)
N3	0.0645 (10)	0.0658 (11)	0.0455 (8)	−0.0144 (9)	−0.0061 (7)	0.0078 (7)
C7	0.0654 (12)	0.0525 (12)	0.0488 (10)	0.0008 (10)	0.0009 (9)	0.0059 (8)
C10	0.0551 (11)	0.0631 (14)	0.0563 (11)	−0.0121 (10)	−0.0008 (9)	0.0036 (9)
C9	0.0572 (12)	0.0670 (15)	0.0611 (12)	−0.0059 (10)	−0.0120 (10)	−0.0078 (10)
C11	0.0885 (16)	0.0861 (19)	0.0494 (12)	0.0190 (14)	−0.0140 (11)	−0.0061 (11)
C2	0.0977 (18)	0.0733 (17)	0.0569 (13)	−0.0359 (15)	−0.0120 (12)	0.0062 (11)
C1	0.0677 (16)	0.095 (2)	0.099 (2)	−0.0246 (15)	−0.0174 (14)	0.0220 (15)

### Geometric parameters (Å, °)

**Table:**

S1—C3	1.6858 (18)	N3—C2	1.462 (3)
N2—C3	1.351 (2)	N3—H3A	0.8600
N2—N1	1.372 (2)	C7—H7A	0.9300
N2—H2A	0.8600	C10—C9	1.381 (3)
N1—C4	1.274 (2)	C10—H10A	0.9300
C5—C10	1.388 (3)	C9—H9A	0.9300
C5—C6	1.390 (3)	C11—H11A	0.9600
C5—C4	1.455 (2)	C11—H11B	0.9600
C6—C7	1.374 (3)	C11—H11C	0.9600
C6—H6A	0.9300	C2—C1	1.502 (4)
C8—C9	1.375 (3)	C2—H2B	0.9700
C8—C7	1.381 (3)	C2—H2C	0.9700
C8—C11	1.508 (3)	C1—H1B	0.9600
C4—H4A	0.9300	C1—H1C	0.9600
C3—N3	1.328 (3)	C1—H1D	0.9600
			
C3—N2—N1	119.36 (16)	C9—C10—C5	120.4 (2)
C3—N2—H2A	120.3	C9—C10—H10A	119.8
N1—N2—H2A	120.3	C5—C10—H10A	119.8
C4—N1—N2	116.63 (16)	C8—C9—C10	121.75 (19)
C10—C5—C6	118.08 (17)	C8—C9—H9A	119.1
C10—C5—C4	119.85 (18)	C10—C9—H9A	119.1
C6—C5—C4	122.08 (17)	C8—C11—H11A	109.5
C7—C6—C5	120.48 (18)	C8—C11—H11B	109.5
C7—C6—H6A	119.8	H11A—C11—H11B	109.5
C5—C6—H6A	119.8	C8—C11—H11C	109.5
C9—C8—C7	117.52 (18)	H11A—C11—H11C	109.5
C9—C8—C11	121.8 (2)	H11B—C11—H11C	109.5
C7—C8—C11	120.7 (2)	N3—C2—C1	111.8 (2)
N1—C4—C5	121.40 (18)	N3—C2—H2B	109.3
N1—C4—H4A	119.3	C1—C2—H2B	109.3
C5—C4—H4A	119.3	N3—C2—H2C	109.3
N3—C3—N2	115.95 (16)	C1—C2—H2C	109.3
N3—C3—S1	124.73 (15)	H2B—C2—H2C	107.9
N2—C3—S1	119.30 (15)	C2—C1—H1B	109.5
C3—N3—C2	125.12 (17)	C2—C1—H1C	109.5
C3—N3—H3A	117.4	H1B—C1—H1C	109.5
C2—N3—H3A	117.4	C2—C1—H1D	109.5
C6—C7—C8	121.8 (2)	H1B—C1—H1D	109.5
C6—C7—H7A	119.1	H1C—C1—H1D	109.5
C8—C7—H7A	119.1		

### Hydrogen-bond geometry (Å, °)

**Table:**

*D*—H···*A*	*D*—H	H···*A*	*D*···*A*	*D*—H···*A*
N2—H2A···S1^i^	0.86	2.69	3.505 (2)	158
N3—H3A···N1	0.86	2.21	2.605 (2)	108

Symmetry codes: (i) −*x*, −*y*+1, −*z*.
